# Utilizing CD44v6 and V600EBRAF-mutation for in vitro targeted combination therapy of thyroid carcinomas

**DOI:** 10.1016/j.heliyon.2023.e22594

**Published:** 2023-11-20

**Authors:** A.C.L. Mortensen, J. Imgenberg-Kreuz, D. Spiegelberg, J. Botling, M. Nestor

**Affiliations:** a Department of Immunology, Genetics and Pathology, Science for Life Laboratory, Uppsala University, Sweden; b Department of Medical Sciences, Uppsala University, Sweden; c Department of Surgical Sciences, Uppsala University, Sweden; d Department of Laboratory Medicine, Institute of Biomedicine, University of Gothenburg, Sweden; e Department of Molecular Medicine and Surgery, Karolinska Institutet, Stockholm, Sweden

**Keywords:** CD44v6, BRAF, Combination therapy, Thyroid cancer, Anaplastic thyroid cancer, targeted therapy, molecular radiotherapy

## Abstract

**Aim:**

The aim of this study was to assess the feasibility of targeted therapy of thyroid carcinoma, first exploring potential targets BRAF, EGFR and CD44v6 in patient material through immunohistochemistry and mutation analysis.

**Materials and methods:**

A patient cohort (n = 22) consisting of seven papillary (PTC), eight anaplastic (ATC) and seven follicular (FTC) thyroid carcinomas were evaluated. Additionally, eight thyroid carcinoma cells lines were analyzed for CD44v6-expression and sensitivity to the multi-kinase inhibitor sorafenib (Nexavar®), which targets numerous serine/threonine and tyrosine kinases, including the Raf family kinases. Targeted therapy using ^131^I-AbN44v6, a novel anti-CD44v6 antibody, and/or sorafenib was evaluated in 3D multicellular tumor spheroids.

**Results:**

Of the two cell surface proteins, EGFR and CD44v6, the latter was overexpressed in >80 % of samples, while EGFR-expression levels were moderate at best in only a few samples. BRAF mutations were more common in PTC patient samples than in ATC samples, while FTC samples did not harbor BRAF mutations. CD44v6-expression levels in the thyroid carcinoma cell lines were more heterogenous compared to patient samples, while BRAF mutational status was in line with the original tumor type. Monotherapy in 3D multicellular ATC tumor spheroids with either ^131^I-AbN44v6 or sorafenib resulted in delayed spheroid growth. The combination of ^131^I-AbN44v6 and sorafenib was the most potent and resulted in significantly impaired spheroid growth.

**Conclusion:**

This “proof of concept” targeted therapy study in the in vitro ATC 3D multicellular tumor spheroids indicated applicability of utilizing CD44v6 for molecular radiotherapy both as a monotherapy and in combination with sorafenib.

## 1 Introduction

Thyroid carcinoma is the most common endocrine malignancy, accounting for up to 4 % of all cancers [1]. The majority of thyroid cancers originate from follicular cells, eliciting well-differentiated thyroid cancers (papillary (PTC) and follicular (FTC)), poorly differentiated thyroid cancers (PDTC) and anaplastic thyroid cancer (ATC) [2,3]. The overall survival rates 40 years post diagnosis for non-advanced, well-differentiated thyroid cancer are excellent (upwards of 95 %). However, PDTC and ATC have a much poorer diagnosis [4]. The five-year-survival rate of PDTC is approximately 20 %, while the median overall survival of ATC is only four-to-six months post diagnosis and the one-year survival falls below 10 % [[5], [6], [7]]. The majority of well-differentiated thyroid cancers are treated successfully with surgery, thyroid hormone suppression therapy and radioactive iodine (RAI) [8]. However, up to one third of patients with advanced disease are resistant to RAI, limiting treatment options [[9], [10], [11]].

The lack of efficacy of conventional therapies for advanced thyroid cancers has resulted in several FDA and EMA recently approved targeted therapies. There are currently three targeted therapies, all of which are tyrosine kinase inhibitors, approved for advanced thyroid cancer: Nexavar® (sorafenib), Lenvima® (lenvatinib) and, most recently, Cabometyx® (cabozantinib). All three are multi-kinases, thus inhibiting a broad range of tyrosine and serine/threonine kinases to varying degrees [[12], [13], [14]]. Sorafenib is of specific interest in thyroid cancer as it targets the Raf-family kinases (RAF-1 and BRAF) in addition to VEGFR, PDGFR and RET [12]. BRAF is a cytoplasmic serine-threonine protein kinase and commonly mutated in thyroid cancer and mutations in BRAF result in constitutive activation, thus promoting proliferation, tumorigenicity and dedifferentiation [[15], [16], [17]].

RAI is considered highly beneficial for treatment of well-differentiated thyroid cancers and is essential in gaining control of metastatic disease. In addition, targeted methods such molecular radiotherapy may be of interest in advanced thyroid cancer. Molecular radiotherapy utilizes a cancer-targeting molecule labeled with a therapeutic radionuclide and delivers a radioactive payload directly to cancer cells, creating a localized radiotherapy. The success of molecular radiotherapy with ^177^Lu-DOTATATE (Lutathera®) and ^177^Lu-PSMA-617 (Pluvicto®) has led to FDA and EMA approvals for the treatment of somatostatin receptor positive neuroendocrine tumors and metastatic castration-resistant prostate cancer, respectively [18]. However, in order for molecular radiotherapy to be applicable for thyroid cancer, new targets need to be identified and explored. One plausible target is the cell surface antigen, CD44v6, which has previously been evaluated as a target for molecular radiotherapy in head and neck squamous cell carcinomas [19]. CD44v6 is overexpressed in a variety of cancers and is often associated with poor prognosis and a more aggressive disease [20,21]. To date, knowledge on CD44v6 expression in thyroid cancer is limited, but a few reports suggest a generally high extent of CD44v6 positive malignancies [22,23]. Thus, utilizing CD44v6 for molecular radiotherapy may be a plausible strategy in advanced thyroid cancer.

Overexpression of EGFR has been documented to a varying degree in thyroid cancer in previous studies [24,25]. When present, EGFR overexpression correlates with a more aggressive disease, particularly in PTC [24]. However, there are conflicting results as to the level and frequency of EGFR overexpression in thyroid cancer and the extent of which the overexpression is associated with aggressive disease and poor prognosis [26]. These conflicting data call for further investigation into the expression levels of EGFR in thyroid cancers to establish whether this could be a plausible target for EGFR targeted therapy.

The aim of this study was to assess the feasibility of targeted radionuclide therapy of thyroid carcinoma, first exploring potential targets BRAF, EGFR and CD44v6 in patient material through immunohistochemistry (IHC) and pyrosequencing. CD44v6-expression levels of both commercially and non-commercially available thyroid cancer cell lines and their sensitivity to sorafenib was evaluated. Finally, a proof of principle therapy study in an in vitro ATC 3D multicellular spheroid model system assessed the applicability of utilizing CD44v6 as a target for molecular radiotherapy both as a monotherapy and in combination with sorafenib.

## 2 Materials & methods

### 2.1 Selection of thyroid cancer tissues

The study was reviewed by the regional ethical committee (Regionala Etikprövningsnämnden Uppsala, Dnr. 2012/342) and no ethical objections were noted regarding the study. Thus, the Uppsala Biobank was explored for tissue samples from papillary, anaplastic and follicular thyroid carcinoma. All specimens were reviewed by a histopathologist (JB). Haematoxylin-eosin stained tissue sections were prepared from formalin-fixed paraffin-embedded (FFPE) tissue blocks (Instrumedics, Richmond, IL). Only tumor tissue sections containing more than 40 % tumor cells were included for molecular analysis. Based on the above-mentioned criteria, tumor tissue blocks were retrieved from 22 patients and selected for analysis; seven PTC, eight ATC and seven FTC.

### 2.2 Immunohistochemical stainings (CD44v6, EGFR, Ki67)

Four-micrometer sections were cut from FFPE-blocks, mounted on adhesive slides and baked at 60 °C for 45 min. The slides were then deparaffinized in xylene, followed by hydration in graded alcohols and blocking for endogenous peroxidase in 0.3 % hydrogen peroxide. For antigen retrieval, a pressure boiler (Decloaking chamber®, Biocare Medical, USA) was used, boiling the slides for 4 min at 125 °C in Target Retrieval Solution (Dako, Denmark). Automated immunohistochemistry (IHC) was performed using an Autostainer XL ST5010 (Leica Microsystems GmbH, Germany). For CD44v6 staining, a dilution of 1:500 of the primary CD44v6-antibody was used (Ab78960, clone VFF-18, Abcam, UK). For EGFR staining, a dilution of 1:100 of the primary antibody was used (article no. 3167, Invitrogen Life Technologies, Sweden). The antibodies were incubated for 30 min at room temperature (RT). The slides were further incubated with the secondary reagent anti-rabbit/mouse horse reddish peroxidase-conjugated UltraVision (Thermo Fischer Scientific) for 30 min at RT. Following washing steps, the slides were developed for 10 min, using diaminobenzidine as a chromogen, and counterstained with Mayer's haematoxylin for 5 min (Sigma-Aldrich, USA). For Ki-67, sections were incubated with an anti-Ki-67 antibody (DakoCytomation) diluted in antibody diluent (DakoCytomation), at room temperature for 60 min. The reaction product was revealed using Dako kit 50087 (DakoCytomation). Sections were counterstained with Mayer's haematoxylin. A number of cells were counted and the Ki-67 index was given as a percentage of positive cells. The slides were then mounted with Pertex® (Histolab AB, Sweden) and scanned using the Aperio ScanScope XT for generation of high-resolution digital images. The intensity of the staining was determined in a graded scale of either negative (0), weak (1), moderate (2) or strong (3). The frequency of stained cells was likewise determined using a graded scale of 0 (0 %), 1 (<30 %), 2 (30–60 %) or 3 (>60 %).

### 2.3 DNA extraction and BRAF pyrosequencing

Genomic DNA was extracted from FFPE tissue sections (10 μm) using the QIAamp DNA Mini Kit (Qiagen GmbH, Hilden, Germany) according to the manufacturer's instructions. The purity and concentration of the extracted DNA was assessed using a NanoDrop instrument (Thermo Scientific, Wilmington, DE).

The PyroMark Q24 BRAF assay (Qiagen) was used to detect mutations in BRAF (codon 600) according to the manufacturer's instructions [27]. PCR primers and sequencing primers were designed using the PyroMark Assay Design 2.0 software (Qiagen). Briefly, ten ng of genomic DNA was used in 25 μL PCR reactions. Twenty μL of the PCR product was subsequently subjected to pyrosequencing using Streptavidin Sepharose High Performance (GE Healthcare, Uppsala, Sweden), PyroMark Gold Q96 reagents, PyroMark Q24 1.0.9 software, and a Q24 instrument (QIAGEN). All identified mutations were confirmed in a second analysis.

### 2.4 Cell lines

The cell line 8305c was purchased from Sigma Aldrich (Darmstadt, Germany) and cultured in Eagle's MEM with 10 % fetal bovine serum (FBS). CAL-62, B-CPAP and 8505c were purchased from DSMZ (Deutsche Sammlung von Mikroorganismen und Zellkulturen GmbH, Braumschweig, Germany) and cultured in Dulbecco's MEM (CAL-62) and RPMI (B-CPAP and 8505c) with 10 % FBS. SW1736 was purchased from CLS Cell line service GmbH (Eppelheim, Germany) and cultured in cultured in RPMI 1640 medium with 10 % FBS. MDA-T32 was purchased from ATCC (American Type Culture Collection, Manassas, VA, USA) and cultured in RPMI 1640 (Biowest, Nuaillé, France) with 10 % FBS. FTC-238 was kindly provided by Dr. Christofer Juhlin at Karolinska Institutet (Stockholm, Sweden) and cultured in Dulbecco's MEM/Ham's F12 (50:50) with 5 % FBS. The ACT-1 cell line was originally established by Dr. Seiji Ohato of Tokushima University [28]. In addition to FBS, all cell medium containing 2 mM l-glutamine and 1 % antibiotics (100 IU penicillin and 100 μg/mL streptomycin). 8305c, 8505c and MDA-T32 were supplemented with 1 % non-essential amino acids. All additives were acquired from Biochrom Kg, Berlin, Germany. All cells were incubated at 37 °C in an atmosphere containing 5 % CO_2_.

### 2.5 Antibody and radiolabeling

The selection, production and specificity of the AbN44v6 antibody has been described previously [29]. Direct iodination of AbN44v6 with ^131^I was performed by adding 20–40 μL (4 mg/mL) Chloramine-T (CAT) to typically 24–120 μg of AbN44v6 (1.2 mg/mL in PBS) and between 2 and 12 MBq of ^131^I with a 60 s incubation on ice before ending the reacting by adding 40–80 μL (4 mg/mL) of Na_2_SO_5_ (NBS). Iodination with ^125^I was done using the Pierce Iodination Method using Pierce iodination tubes per manufacturer's instructions. Typically, 50 μg of AbN44v6 (1.2 mg/mL in PBS) were labeled with 5 MBq of ^125^I. Instant thin layer chromatography (ITLC) determined the labeling yield using 70 % acetone as the mobile phase and a Bass 1800 II phosphoimaging system (Fuji, Japan) for analysis. The labeling yields of ^131^I-AbN44v6 were 88 % ± 7 %, with specific activities of 73 kBq ± 6 kBq per μg. The yields of all ^125^I-iodinations were 100 %, with specific activities of 100 kBq/μg.

### 2.6 LigandTracer

Experiments were performed as described previously [30]. Typically, 10^6^ cells were seeded per dish of each cell line and incubated at 37 °C and 5 % CO_2_ for at least 24 h prior to the start of experiments. Concentrations of 3 nM and 10 nM of ^131^I-AbN44v6 and incubated for at least 90 min each followed by a dissociation phase of at least 12 h.

### 2.7 Cell viability assays, 2,3-Bis(2-methoxy-4-nitro-5-sulfophenyl)-2H-tetrazolium-5-carboxanilide salt (XTT)

Cells were seeded in flat-bottomed 96-well plates and incubated for 48 h prior to drug incubation. For drug incubation, 0–30 μM of sorafenib was incubated for 72 h (Selleckchem, USA). Cell viability was measured according to manufacturer's instructions of the ATCC Cell proliferation Assay Kit (ATCC, Manassas, VA, USA) using an iMark™ Microplate reader (Bio-Rad, Hercules, CA, USA). Experiments were optimized for cell count and repeated at least three times.

### 2.8 Estimated CD44v6 expression levels

Cells were seeded in 48-well plates and incubated for at least 24 h prior to the start of experiments. Cells were incubated for 24 h with either 10 nM ^125^I-AbN44v6 or 10 nM ^125^I-AbN44v6 with a 100-fold molar excess of non-radiolabeled AbN44v6. After 24 h, the incubation medium was removed and the wells were washed at least four times with PBS prior to harvesting and cell counting. Samples were measured in a Wizard 2460 automated well-counter (PerkinElmer, Massachusetts, USA). The counts per minute (CPM) were calculated per 10^5^ cells, where background signal, defined as signal obtained in the presence of 1 μM unlabeled antibody, was deducted from the amassed CPM.

### 2.9 3D multicellular tumor spheroids

For spheroid formation, 8305C cells were seeded in flat-bottomed 96-well plates pre-coated with agarose as previously described and incubated for 72 h prior to start of treatment [31]. Spheroids were subsequently treated with either 0–90 kBq (30 nM) of ^131^I-AbN44v6, 90 kBq of free ^131^I or 4.4 μM sorafenib as well as the combination of sorafenib and ^131^I-AbN44v6. Pictures were obtained at the start of treatment and every three-to-four days using a Canon EOS 700D camera mounted on an inverted Nikon Diaphot-TMD microscope. Half of the incubation medium was replaced at each time point. Spheroid volume was determined through measuring the surface area using ImageJ software, version 1.48 (NIH, Bethesda, MD, USA) and calculating the corresponding volume, assuming the spheroids retained a spherical form using the formula: 4/3π*r^3^.

### 2.10 8305c and ACT-1 xenografts and ex vivo IHC

Three female Balb/c nu/nu mice (6 weeks old, approximately 20 g) were housed under standard laboratory conditions and ad libitum access to food and water. All experiments complied with current Swedish law and were performed with permission granted by the Uppsala Committee of Animal Research Ethics (C33/16). Approximately 10^7^ 8305c or ACT-1 cells were inoculated on each flank in serum-free medium. After an incubation period of two-to-three weeks, the mice were euthanized by injection of a mixture of ketamine and xylazine followed by heart puncture. 8305c and ACT-1 xenografts were fixated in formalin and subsequently paraffin-embedded, sectioned and deparaffinized. Sections were immunostained with an anti-CD44v6 antibody as described above for patient IHC.

### 2.11 Statistical analyses

Graphpad Prism version 6.07 (Graphpad Software, San Diego, CA, USA) was used for data processing and analysis. Significance was determined using one-way ANOVA followed by Tukey's multiple comparisons test, with p < 0.05 (*), p < 0.01 (**), p < 0.001 (***), p < 0.0001 (****). A version of the Valeriote and Lin model was applied for calculation of expected additive combination effects of spheroids and has been described previously [32]. In short, effects of the combination of sorafenib and ^131^I-AbN44v6 were analyzed, using a version of the ‘additive model’. The survival fraction (SF) after treatment with either sorafenib or ^131^I-AbN44v6 were multiplied to each other to form an ‘expected value’ of additive SF (SF_exp.add_). When the observed SF after combined treatment was significantly lower than the expected additive SF, SF_obs_ < SF_exp.add_, the response was defined as potentiating.

## 3 Results

### 3.1 Immunohistochemistry and mutation analysis of patient samples

**Fig. 1:**
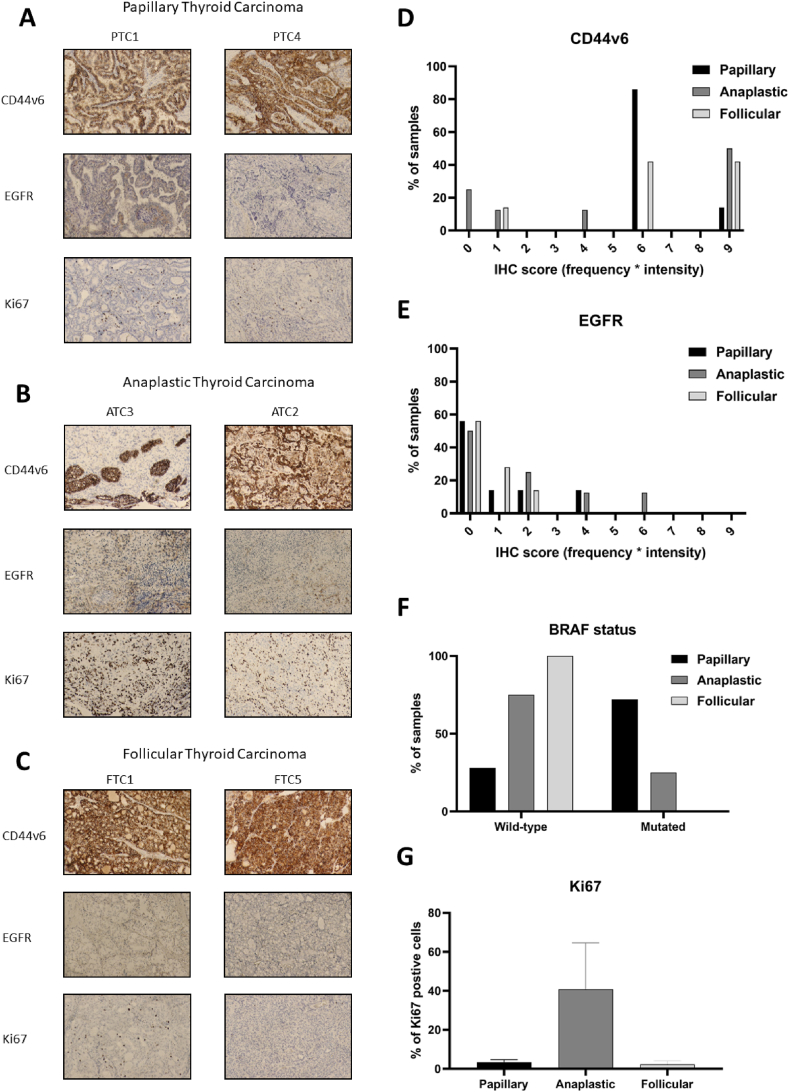
Representative examples of IHC staining of CD44v6 expression, EGFR expression and Ki67 of A) two papillary thyroid carcinoma patient samples (PTC2 and PTC4), B) two anaplastic thyroid carcinoma patient samples (ATC3 and ATC5), and C) two follicular thyroid carcinoma patient samples (FTC5 and FTC1). D) IHC scoring (presented as frequency x intensity) of CD44v6 for all investigated patient samples, E) IHC scoring of EGFR (presented as frequency x intensity) for all investigated patient samples, F) BRAF status for all investigated patient samples, and G) percentage Ki67 positive cells for all investigated patient samples. Images presented as 10× magnification.

In order to assess suitable targets for molecular radiotherapy in thyroid cancer, stainings were performed on patient material from seven PTC, eight ATC, and seven FTC patients. Examples of IHC stainings can be seen in Fig. 1A–C, and the complete list of evaluation results can be seen in Table 1, Table 2, Table 3. Stronger stainings were observed for CD44v6 than EGFR for all three thyroid cancer types (Fig. 1D and E, Table 1, Table 2, Table 3). 100 % of PTC and 50 % and 86 % of ATC and FTC respectively demonstrated an IHC score (frequency * intensity) or six or more for CD44v6 stainings. For EGFR stainings, none of the PTCs or FTCs and only one of the ATCs (14 %) demonstrated an IHC score of six. No correlation between CD44v6 expression and EGFR expression was seen, nor between BRAF-status and CD44v6 or EGFR expression. As expected, ATC stood out in Ki67 scores, with 41 ± 8 % (SEM) of positive cells, compared with only 3 ± 0.5 % and 2 ± 0.7 % for papillary and follicular cancer respectively (Fig. 1G). BRAF mutations were most frequent in PTC (71 %), followed by ATC (25 %). No BRAF mutations were detected in the FTC samples (Fig. 1F).

**Table 1:** Assessment of seven papillary thyroid carcinoma patient samples as measured in frequency x intensity of CD44v6 and EGFR expression, BRAF status and % positive cells for proliferation marker Ki67.

Type	Block	BRAF	CD44v6		EGFR		Ki67 (%)
			Frequency	Intensity	Frequency	Intensity	
*Papillary*	PTC1	mut	3	2	2	2	5
	PTC2	mut	3	2	2	1	1
	PTC3	mut	3	2	1	1	3
	PTC4	mut	3	2	0	0	4
	PTC5	wt	3	3	0	0	4
	PTC6	wt	3	2	0	0	3
	PTC7	mut	3	2	0	0	4

**Table 2:** Assessment of eight ATC patient samples as measured in frequency x intensity of CD44v6 and EGFR expression, BRAF status and % positive cells for proliferation marker Ki67.

Type	Block	BRAF	CD44v6		EGFR		Ki67 (%)
			Frequency	Intensity	Frequency	Intensity	
*Anaplastic*	ATC1	wt	3	3	2	1	60
	ATC2	wt	3	3	2	2	70
	ATC3	wt	3	3	2	1	60
	ATC4	wt	2	2	0	0	12
	ATC5	mut	3	3	3	2	30
	ATC6	wt	1	1	0	0	40
	ATC7	wt	0	0	0	0	4
	ATC8	mut	0	0	0	0	50

**Table 3:** Assessment of seven FTC patient samples as measured in frequency x intensity of CD44v6 and EGFR expression, BRAF status and % positive cells for proliferation marker Ki67.

Type	Block	BRAF	CD44v6		EGFR		Ki67 (%)
			Frequency	Intensity	Frequency	Intensity	
*Follicular*	FTC1	wt	3	3	1	1	6
	FTC2	wt	3	2	0	0	1
	FTC3	wt	3	2	1	2	1
	FTC4	wt	1	1	0	0	2
	FTC5	wt	3	3	0	0	3
	FTC6	wt	3	2	0	0	2
	FTC7	wt	3	3	1	1	1

### 3.2 Evaluation of CD44v6-antigen expression levels, uptake and retention of ^131^I-AbN44v6

**Fig. 2:**
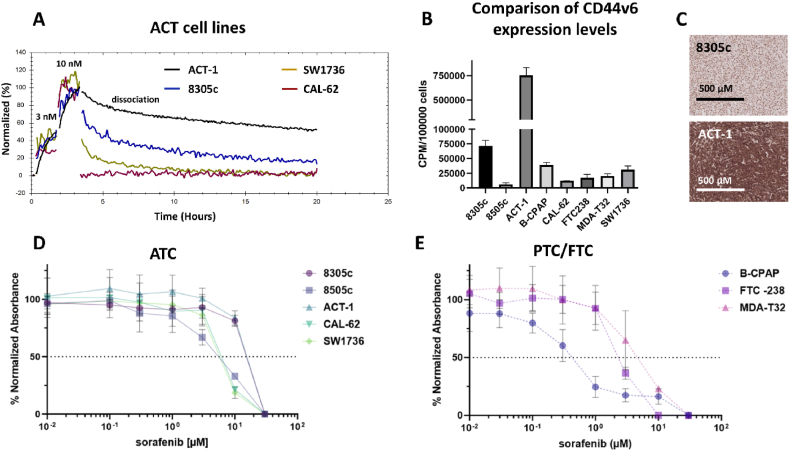
A) LigandTracer evaluation of CD44v6-binding with 3 nM and 10 nM of ^131^I-AbN44v6 on ATC cell lines ACT-1, 8305c, SW1736 and CAL-62, followed by retention measurements (0 nM). B) CD44v6-antigen expression levels as CPM/100000 cells of ATC/PTC/FTC cell lines. C) IHC representative image of the CD44v6-antigen expression of an 8305c and an ACT-1 xenograft. D) Representative viability of ATC cell lines and E) Representative viability of PTC and FTC cell lines incubated with 0.01–30 μM sorafenib for 72 h normalized to viability of untreated controls. Error bars represent SD, n ≥ 6. Images presented at 10× magnification.

A panel of eight thyroid cancer cell lines (Table 4) were evaluated for CD44v6 expression though assessment of cellular uptake of an iodinated antibody targeting CD44v6, ^125^I-AbN44v6. Half of the cell lines demonstrated measurable expression levels, whereas the remaining 50 % demonstrated low-to-negative levels (Fig. 2B). The ACT-1 cells demonstrated the highest uptake of AbN44v6 in all cell-based assays and IHC on tumor xenografts confirmed a high presence of CD44v6 (Fig. 2C). Of the additional cell lines, the highest antigen expression level was detected in the 8305c and IHC of tumor xenografts confirmed the low-to-moderate presence of CD44v6 (Fig. 2C). LigandTracer evaluation confirmed binding and retention of ^131^I-AbN44v6 on the ACT-1, 8305c and SW1736 cells, but not the CAL-62 cells, indicating that the antigen expression level of CAL-62 was below the detection limit of the LigandTracer system (Fig. 2A).

**Table 4:** Table of cell lines utilized in this study: their cancer origin, published BRAF-status and estimated sorafenib IC50 values, presented as averages of triplicate experiments, as measured through XTT-viability following 72 h incubation with 0.01–30 μM of sorafenib. Wild-type written as wt.

Cell line	Cancer origin	BRAF status	Sorafenib IC_50_
8305c	ATC	V600E [16]	12 μM
8505c	ATC	V600E [16]	5.3 μM
ACT-1	ATC	wt (NRAS mutant) [16]	15 μM
B-CPAP	PTC	V600E [33]	0.4 μM
CAL-62	ATC	wt (KRAS mutant) [34]	9 μM
FTC-238	FTC	wt [35]	6.5 μM
MDA-T32	PTC	V600E [36]	4.5 μM
SW1736	ATC	V600E [15]	8.1 μM

### 3.3 Sorafenib sensitivity

The sensitivity of eight thyroid cancer cell lines to sorafenib was assessed using 2D viability assays (XTT). IC_50_ values were determined from repeated assays and the average values can be found in Table 4. Among the evaluated cell lines, ACT-1 (BRAF wt) was the most resistant to sorafenib, while B-CPAP (BRAF^V600E^) was the most sensitive (Fig. 2D and E, Table 4). Of the cell lines harboring the BRAF^V600E^, 8305c was the most resistant with an IC_50_ of 12 μM following the 72 h drug incubation. The remaining cell lines were within a relatively narrow range of sensitivity (4.5–9 μM) irrespective of BRAF mutational status.

### 3.4 3D in vitro molecular radiotherapy and combination therapy

**Fig. 3:**
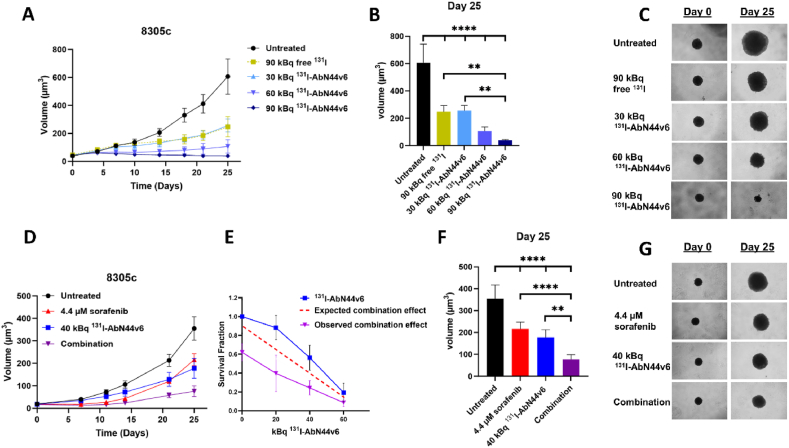
A) Volumes of 8305c 3D multicellular tumor spheroids following treatment with 0–90 kBq of ^131^I-AbN44v6 or 90 kBq of free ^131^I. B) Corresponding One-way Anova on Day 25 post treatment of 8305c spheroids. C) Representative images of treatments at start of treatment (day 0) and end of assay (day 25). D) Volume of 8305c 3D multicellular tumor spheroids following treatment with 4.4 μM sorafenib, 40 kBq of ^131^I-AbN44v6 or the combination of the two monotherapies. E) Expected and observed combination effect of ^131^I-AbN44v6 (0, 20, 40 and 60 kBq) and 4.4 μM sorafenib. F) Corresponding one-way Anova on Day 25 post combination treatment of 8305c spheroids. G) Representative images at start of treatment (day 0) and end of assay (day 25). n ≥ 4, error bars represent 95 % confidence intervals.

The potential for molecular radiotherapy of CD44v6-targeted ^131^I-labeled antibodies was then further assessed in a 3D multicellular spheroid model using 8305c spheroids, a BRAF^V600E^ cell line with a moderate expression level of CD44v6. All tested activities of ^131^I-AbN44v6 had a significant inhibitory impact on the growth of 8305c spheroids compared to untreated controls (Fig. 3). A three-fold increase in activity (90 kBq) of free ^131^I was needed in order to obtain equivalent growth inhibition compared to targeted activity using ^131^I-AbN44v6 (30 kBq). Comparatively, 90 kBq of ^131^I-AbN44v6 resulted in spheroid regression. Both monotherapies (40 kBq ^131^I-AbN44v6 and 4.4 μM sorafenib) and the combination therapy resulted in significant growth inhibition compared to untreated controls (p < 0.0001) by day 25 (Fig. 3B and F). The growth inhibition of the combination therapy was superior to both sorafenib (p < 0.0001) and ^131^I-AbN44v6 (p < 0.01) monotherapies. Similarly, the observed combination effect was greater than the calculated, expected effect (Fig. 3E).

## 4 Discussion

The aim of this study was to explore EGFR, CD44v6 and BRAF as molecular targets in thyroid cancer by assessing patient samples from PFC, FTC and ATC, as well as in cultured thyroid cancer cell lines. The most promising targets were explored with a proof-of-principle therapeutic 3D therapy study in vitro, using sorafenib and a CD44v6-targeting antibody labeled with ^131^I.

EGFR-targeted treatment has been suggested as a treatment option in thyroid cancer, although the EGFR overexpression has not been widely investigated [25]. In general, overexpression of EGFR is associated with a more aggressive disease, yet according to Jankovic et al. (2017), EGFR-overexpression is correlated to BRAF wt-status and is absent to a greater extent in the more aggressive thyroid cancer subtypes [24,26]. This is in line with studies that have shown how BRAF mutations are more frequent in aggressive thyroid cancer subtypes [16]. Interestingly, the correlation made by Jankovic et al. between EGFR-overexpression and BRAF wt-status does not correspond with patient data presented in our study. The single case with higher EGFR expression (IHC score of 6) in our data was an ATC carrying a BRAF mutation. Overall, our results suggest that EGFR expression levels are generally low in thyroid cancer, indicating that EGFR might be a poor therapeutic target for this patient group. However, the results of our small cohort should not be regarded as conclusive, rather that the subject warrants further studies.

The BRAF status of thyroid cancer patients is of great importance, both in terms of prognosis and treatment plan [37,38]. BRAF mutations are frequent among PTC, whereas the MAPK pathway is commonly disrupted in FTC through RAS-mutations [39,40]. Our data indicate that BRAF is frequently mutated in PTC, to a lesser extent in ATC (Fig. 1F) and absent in FTC. These results are all in line with current research [[39], [40], [41]]. BRAF remains an important target in PTC and ATC and a new generation of highly selective BRAF-inhibitors such as dabrafenib are making their mark [42].

The greater frequency of BRAF mutation among the commercially available ATC cell lines could indicate that the majority are PTC derived [41]. The sensitivity of the investigated cell lines to sorafenib treatment did not correlate with BRAF mutational status (Fig. 2D and E, Table 4). The two most sensitive cell lines in this study, 8505c and B-CPAP, as well as one of most resistant cell lines, 8305c, are all BRAF^V600E^ mutants. Resistance to sorafenib despite BRAF^V600E^ mutations is common and has been reported in previous studies [15,43].

The CD44v6 expression in thyroid cancers has not been widely studied. However, a few reports present clinical data indicating that CD44v6 is present to a great extent in both PTC and FTC [23,44]. Wu et al. (2013) stained 35 PTC primary tumors and found that 80 % were positive for CD44v6 [23]. This correlates with our data, where all assessed PTC samples were positive for CD44v6 (Fig. 1, Table 1). Overall, the CD44v6 expression levels in ATC patient samples were more dispersed than in PTC and FTC, albeit 50 % were high expressing with an IHC score of 9 (Fig. 1, Table 2). The dispersed CD44v6 expression pattern in the patient material was mirrored in the evaluated ATC cell lines. Among the six ATC cell lines, 50 % demonstrated clear CD44v6 expression (ACT-1, 8305c, SW1736), while the remaining half were low expressing or lacking CD44v6 expression (Fig. 2B and C). In 3D multi-cellular tumor spheroid assays, 8305c spheroids responded in an activity-dose-dependent manner to targeted molecular radiotherapy with ^131^I-AbN44v6 (Fig. 3A–C). Spheroids treated with 90 kBq ^131^I-AbN44v6 resulted in complete growth arrest and spheroid shrinkage. The presence of free ^131^I (90 kBq) in the cell medium resulted in only limited effects on spheroid growth. The limited effect of free ^131^I on spheroid growth was in line with previous studies, where an unspecific ^131^I-labeled isotope control antibody (i.e., rituximab) was evaluated side-by-side with radiolabeled ^131^I-AbN44v6 [29]. These results validate the antigen-specific effects of targeted molecular radiotherapy in the present study. These potent effects of ^131^I-AbN44v6 are surprising, given the low-to-moderate CD44v6 expression levels of the 8305c cell line. It is likely that CD44v6 is upregulated in the 3D setting and even more so *in vivo* due to changes in access to nutrients and oxygen compared to a 2D setting [45]. The CD44v6 expression in 8305c xenografts confirmed a low-to-moderate expression level, although the levels were considerably lower compared to the ACT-1 xenograft expression level. The 8305c cell line is considered resistant to sorafenib, with demonstrated IC_50_ values as high as 20 μM in 2D cell-based assays [46]. The sorafenib dose administered in the spheroid study (4.4 μM) was deliberately below the measured IC_50_ (12 μM) to ensure a moderate response to monotherapy. Interestingly, the combination of ^131^I-AbN44v6 (40 kBq) and sorafenib (4.4 μM) resulted in significant inhibition of spheroid growth compared to monotherapies and controls (Fig. 3D, 3F-G). Additionally, the observed combination effect was superior to the expected effect (Fig. 3E), indicating a clear potentiating effect and further highlighting the potency of the combination. This proof-of-principle combination of ^131^I-AbN44v6 and sorafenib in 8305c 3D multicellular tumor spheroids demonstrated how the combination of two targeted therapies can successfully lower the dosages while increasing efficacy.

## 5 Conclusion

CD44v6 is a clinically validated target for molecular radiotherapy [47]. Given the high frequency and intensity of the expression levels in patient samples of PTC, ATC and FTC presented here, utilizing CD44v6 as a target for molecular radiotherapy against advanced thyroid cancers could potentially be highly beneficial. The possibility of combining CD44v6-targeted molecular radiotherapy with multi-kinase TKIs such as sorafenib or newer, more specific inhibitors such as dabrafenib and trametinib, poses an interesting therapeutic option that warrants further studies.

## Funding

This work was supported by the 10.13039/501100002794Swedish Cancer Society (Cancerfonden) through multiple grants, the 10.13039/501100003748Swedish Society for Medical Research (SSMF) and the Swedish Scientific Council (Vetenskapsrådet).

## Disclosure statement

The authors declare no conflict of interest.

## Declarations

The patient data collection and subsequent study was reviewed by the regional ethical committee (Regionala Etikprövningsnämnden Uppsala, Dnr. 2012/342) and no ethical objections were noted regarding the study. All animal experiments complied with current Swedish law and were performed with permission granted by the Uppsala Committee of Animal Research Ethics (C33/16).

## Data availability statement

All data is available on request and has not been published elsewhere. The data was not deposited into a publicly available repository prior to publication due to ongoing patent-pending investigations of a related antibody project.

## CRediT authorship contribution statement

**A.C.L. Mortensen:** Conceptualization, Data curation, Formal analysis, Investigation, Methodology, Project administration, Validation, Visualization, Writing – original draft, Writing – review & editing. **J. Imgenberg-Kreuz:** Data curation, Formal analysis, Methodology, Validation, Writing – review & editing. **D. Spiegelberg:** Data curation, Formal analysis, Investigation, Software, Validation, Visualization, Writing – review & editing. **J. Botling:** Conceptualization, Data curation, Investigation, Methodology, Resources, Supervision, Validation, Visualization, Writing – review & editing. **M. Nestor:** Conceptualization, Data curation, Formal analysis, Funding acquisition, Investigation, Methodology, Project administration, Resources, Supervision, Validation, Writing – original draft, Writing – review & editing.

## Declaration of competing interest

The authors declare that they have no known competing financial interests or personal relationships that could have appeared to influence the work reported in this paper.

## References

[1] Pizzato M. (2022). The epidemiological landscape of thyroid cancer worldwide: GLOBOCAN estimates for incidence and mortality rates in 2020. Lancet Diabetes Endocrinol..

[2] Saini S., Tulla K., Maker A.V., Burman K.D., Prabhakar B.S. (2018). Therapeutic advances in anaplastic thyroid cancer: a current perspective. Mol. Cancer.

[3] Veschi V. (2020). Cancer stem cells in thyroid tumors: from the origin to metastasis. Front. Endocrinol..

[4] Jukic T. (2022). Long-term outcome of differentiated thyroid cancer patients-fifty years of Croatian thyroid disease referral centre experience. Diagnostics.

[5] Maniakas A. (2020). Evaluation of overall survival in patients with anaplastic thyroid carcinoma, 2000-2019. JAMA Oncol..

[6] Abdulghani J. (2013). Sorafenib sensitizes solid tumors to Apo2L/TRAIL and Apo2L/TRAIL receptor agonist antibodies by the Jak2-Stat3-Mcl1 axis. PLoS One.

[7] de Ridder M., Nieveen van Dijkum E., Engelsman A., Kapiteijn E., Klumpen H.J., Rasch C.R.N. (2020). Anaplastic thyroid carcinoma: a nationwide cohort study on incidence, treatment and survival in The Netherlands over 3 decades. Eur. J. Endocrinol..

[8] Stewart L.A., Kuo J.H. (2021). Advancements in the treatment of differentiated thyroid cancer. Ther. Adv. Endocrinol. Metab..

[9] Liu J., Liu Y., Lin Y., Liang J. (2019). Radioactive iodine-refractory differentiated thyroid cancer and redifferentiation therapy. Endocrinol. Metab. (Seoul).

[10] Worden F. (2014). Treatment strategies for radioactive iodine-refractory differentiated thyroid cancer. Ther. Adv. Med. Oncol..

[11] Kersting D. (2021). Predictive factors for RAI-refractory disease and short overall survival in PDTC. Cancers.

[12] Gong L., Giacomini M.M., Giacomini C., Maitland M.L., Altman R.B., Klein T.E. (Jun 2017). PharmGKB summary: sorafenib pathways. Pharmacogenet Genomics.

[13] Grullich C. (2018). Cabozantinib: multi-kinase inhibitor of MET, AXL, RET, and VEGFR2. Recent Results Cancer Res..

[14] Capozzi M. (2019). Lenvatinib, a molecule with versatile application: from preclinical evidence to future development in anti-cancer treatment. Cancer Manag. Res..

[15] Broecker-Preuss M. (2015). Sorafenib inhibits intracellular signaling pathways and induces cell cycle arrest and cell death in thyroid carcinoma cells irrespective of histological origin or BRAF mutational status. BMC Cancer.

[16] Kunstman J.W. (2015). Characterization of the mutational landscape of anaplastic thyroid cancer via whole-exome sequencing. Hum. Mol. Genet..

[17] Croce L., Coperchini F., Magri F., Chiovato L., Rotondi M. (2019). The multifaceted anti-cancer effects of BRAF-inhibitors. Oncotarget.

[18] Sartor O. (2021). Lutetium-177-PSMA-617 for metastatic castration-resistant prostate cancer. N. Engl. J. Med..

[19] Tijink B.M. (2006). A phase I dose escalation study with anti-CD44v6 bivatuzumab mertansine in patients with incurable squamous cell carcinoma of the head and neck or esophagus. Clin. Cancer Res..

[20] Todaro M. (2014). CD44v6 is a marker of constitutive and reprogrammed cancer stem cells driving colon cancer metastasis. Cell Stem Cell.

[21] Wang Y., Yang X., Xian S., Zhang L., Cheng Y. (Jul 2019). CD44v6 may influence ovarian cancer cell invasion and migration by regulating the NF-kappaB pathway. Oncol. Lett..

[22] Okada T. (2014). Coexpression of EpCAM, CD44 variant isoforms and claudin-7 in anaplastic thyroid carcinoma. PLoS One.

[23] Wu G., Zhou Y., Li T., Guo J., Zhou Z. (Jun 2013). Immunohistochemical levels of matrix metalloproteinase-2 and CD44 variant 6 protein in the diagnosis and lateral cervical lymph node metastasis of papillary thyroid carcinoma. J. Int. Med. Res..

[24] Fisher K.E. (Nov 2013). Epidermal growth factor receptor overexpression is a marker for adverse pathologic features in papillary thyroid carcinoma. J. Surg. Res..

[25] Schiff B.A. (2004). Epidermal growth factor receptor (EGFR) is overexpressed in anaplastic thyroid cancer, and the EGFR inhibitor gefitinib inhibits the growth of anaplastic thyroid cancer. Clin. Cancer Res..

[26] Jankovic J., Tatic S., Bozic V., Zivaljevic V., Cvejic D., Paskas S. (Mar 2017). Inverse expression of caveolin-1 and EGFR in thyroid cancer patients. Hum. Pathol..

[27] Birgisson H. (2015). Microsatellite instability and mutations in BRAF and KRAS are significant predictors of disseminated disease in colon cancer. BMC Cancer.

[28] Chung S.H., Onoda N., Ishikawa T., Ogisawa K., Takenaka C., Yano Y., Hato F., Hirakawa K. (2002). Peroxisome proliferator-activated receptor gamma activation induces cell cycle arrest via the p53-independent pathway in human anaplastic thyroid cancer cells. Jpn. J. Cancer Res..

[29] Mortensen A.C., Spiegelberg D., Haylock A.K., Lundqvist H., Nestor M. (Jun 2018). Preclinical evaluation of a novel engineered recombinant human anti-CD44v6 antibody for potential use in radio-immunotherapy. Int. J. Oncol..

[30] Stenberg J., Spiegelberg D., Karlsson H., Nestor M. (Feb 2014). Choice of labeling and cell line influences interactions between the Fab fragment AbD15179 and its target antigen CD44v6. Nucl. Med. Biol..

[31] Mortensen A.C.L., Spiegelberg D., Brown C.J., Lane D.P., Nestor M. (2019). The stapled peptide PM2 stabilizes p53 levels and radiosensitizes wild-type p53 cancer cells. Front. Oncol..

[32] Valeriote F., Lin H. (1975). Synergistic interaction of anticancer agents: a cellular perspective. Cancer Chemother. Rep..

[33] Coperchini F. (2019). The BRAF-inhibitor PLX4720 inhibits CXCL8 secretion in BRAFV600E mutated and normal thyroid cells: a further anti-cancer effect of BRAF-inhibitors. Sci. Rep..

[34] Audrito V. (2022). Tumors carrying BRAF-mutations over-express NAMPT that is genetically amplified and possesses oncogenic properties. J. Transl. Med..

[35] Bonaldi E. (2021). BRAF inhibitors induce feedback activation of RAS pathway in thyroid cancer cells. Int. J. Mol. Sci..

[36] Henderson Y.C. (Feb 2015). Development and characterization of six new human papillary thyroid carcinoma cell lines. J. Clin. Endocrinol. Metab..

[37] Kebebew E. (2007). The prevalence and prognostic value of BRAF mutation in thyroid cancer. Ann. Surg..

[38] Savvides P. (May 2013). Phase II trial of sorafenib in patients with advanced anaplastic carcinoma of the thyroid. Thyroid.

[39] Xing M. (2016). Clinical utility of RAS mutations in thyroid cancer: a blurred picture now emerging clearer. BMC Med..

[40] Howell G.M., Hodak S.P., Yip L. (2013). RAS mutations in thyroid cancer. Oncol..

[41] Zou M. (Aug 2014). Concomitant RAS, RET/PTC, or BRAF mutations in advanced stage of papillary thyroid carcinoma. Thyroid.

[42] Subbiah V. (Apr 2022). Dabrafenib plus trametinib in patients with BRAF V600E-mutant anaplastic thyroid cancer: updated analysis from the phase II ROAR basket study. Ann. Oncol..

[43] Talezadeh Shirazi P. (2022). Eugenol: a new option in combination therapy with sorafenib for the treatment of undifferentiated thyroid cancer. Iran. J. Allergy, Asthma Immunol..

[44] Guan M., Ma Y., Shah S.R., Romano G. (2016). Thyroid malignant neoplasm-associated biomarkers as targets for oncolytic virotherapy. Oncolytic Virother..

[45] Senkowski W. (2016). Large-scale gene expression profiling platform for identification of context-dependent drug responses in multicellular tumor spheroids. Cell Chem. Biol..

[46] Mortensen A.C.L., Berglund H., Hariri M., Papalanis E., Malmberg C., Spiegelberg D. (2023). Combination therapy of tyrosine kinase inhibitor sorafenib with the HSP90 inhibitor onalespib as a novel treatment regimen for thyroid cancer. Sci. Rep..

[47] Borjesson P.K. (2003). Phase I therapy study with (186)Re-labeled humanized monoclonal antibody BIWA 4 (bivatuzumab) in patients with head and neck squamous cell carcinoma. Clin. Cancer Res..

